# Reply to: Coastal shoreline change assessments at global scales

**DOI:** 10.1038/s41467-024-46609-w

**Published:** 2024-03-15

**Authors:** Rafael Almar, Julien Boucharel, Gregoire Ondoa Abessolo, Fabrice Papa, Erwin W. J. Bergsma

**Affiliations:** 1 LEGOS (Université de Toulouse/CNRS/IRD/CNES), Toulouse, France; 2 Department of Atmospheric Sciences (University of Hawaii at Manoa), Honolulu, HI USA; 3 Ecosystems and Fishery Resources Laboratory, Institute of Fisheries and Aquatic Sciences, University of Douala, Douala, Cameroon; 4 Universidade de Brasília (UnB), IRD, Instituto de Geociencias, Brasilia, Brazil; 5 Earth Observation Lab, French Space Agency (CNES), Toulouse, France

**Keywords:** Geomorphology, Climate-change impacts, Physical oceanography

**replying** **to** J. Warrick et al. *Nature Communications* 10.1038/s41467-024-46608-x (2024)

We thank the authors of the Matters Arising manuscript for their interest in our study. We are open to, and cherish the virtues of scientific debate and criticism but we are not sure that we fully understand the authors’ intentions on this matter, as the responses to the issues they raised are actually embedded in our published manuscript. We believe this is due to a misunderstanding of the aim, scope and scale of our study by authors who are mainly from the coastal geomorphology community. The MA indeed mostly deals with short and local scales and sedimentary processes, while our original study positions itself at long-term and interannual timescales, from regional to global scales, emphasizing ocean and climate processes that drive both coastal erosion and flooding. These are aspects that have been overlooked for too long, including by the MA co-authors in their own studies. Thus, beyond a priori and opinionated commentaries, we prefer to encourage data-evidenced science.

With our reply, we highlight again why we believe that global interdisciplinary studies are important for coastal science and what new perspectives are emerging in the era of global satellite observations and climate-based predictions.

## On the choice of a proxy for land–sea interface: the important distinction between a constant-topography level shoreline and waterline

There seems to be a fundamental misunderstanding here by Warrick et al. who refer to morphological changes only and seem to be looking for a geological iso-level shoreline. We explicitly use the “shoreline” position as the land–sea waterline observed by the satellite to, indeed, investigate the influence of all environmental components holistically. We remind here that land–sea measurements reflect both morphological processes (erosion/accretion), including the influence of tides and waves, but also, and more importantly, a large variety of coastal sea level components^1^. Notably, these are complex mechanisms that are yet to be better understood (e.g., steric influence of river freshwater^2^, upwelling, ocean circulation…etc.^3–5^) and are an active line of physical oceanography research. Whereas Warrick et al. refer to sandy open beaches exposed to waves, here we consider all types of coasts, including also closed seas and sheltered coasts where river freshwater flow has an obviously greater influence on the waterline position. Therefore, considering only tide and wave setup instead of all sea level components is a very strong approximation of reality and introduces even large uncertainties in the estimated iso-level shoreline from the satellite. Ocean steric and dynamic variations can reach vertical amplitudes of tens of centimeters up to a meter on seasonal to interannual scales^6^ and should not be excluded anymore: not looking at it, does not mean it does not exist. Our study should be taken like many other investigations, a scientific study that brings new knowledge to understand our coasts with its own limitations. Here in particular, we reveal the key role of SLA at interannual scales, as already mentioned^7^ in the Pacific, an information also useful to better assess iso-level shoreline from satellite.

## Scaling up coastal science: the imperative shift from local to regional climate coastal perspectives

The authors seem to state that global scale studies are useless (and may even be misleading) and only site-specific local scale studies would be useful. In the limitations section, we clearly position our study at climate, annual, and global scales. Local waterline dynamics, in spite of its interest for coastal risks and management, is much more complex than what we capture and focus on in our study. In the manuscript, we recognize that several other methods, including in situ measurements, are more appropriate and accurate for local studies. There is no doubt that detailed, local scale studies are required to adequately inform local scale decisions. However, global studies bring important regional baseline and boundaries for local detailed studies. Global studies are also necessary to identify regional hotspots, drive detailed measurement efforts to inform national or larger-scale sectorial decisions and initiatives that need to be looked more closely using higher resolution models^8^. By analogy, General Circulation Models (GCMs), which are mostly based on 0.5–1 degree resolution and are routinely used by global initiatives such as the Intergovernmental Panel on Climate Change (IPCC), are intended to be an efficient tool for assessing global trends and a first pass for climate variability over large areas (e.g., oceanic basins or the scale of the 44 IPCC AR6 regions, which together cover the entire world), but, for instance, they should be used with caution at the scale of individual cities. Our global study falls in the same category and we maintain that our results are useful to drive agendas and initiatives at higher spatial scales (national, continental and global) such as those done by national governments, United Nations agencies, World Bank…etc.

## The breakthrough of global hindcasts and satellite observations

Instead of having sparse site-specific observations, and in line with previous studies (basin-scale^7^; regional-scale^9^), here our admittedly coarse—0.5°—resolution but continuous approach represents a current best effort for global coverage, while finer transect resolution and spatial averaging are on the horizon. Our novel approach demonstrates the critical coverage and observational capabilities provided by satellites today^10,11^, especially in: (1) regions of the world where field data are essentially lacking, either because of limited research funding and capacity, such as in developing countries (e.g., the African continent --notably under-represented in international studies^12^), and (2) where logistical or accessibility problems simply prohibit or bias (calm conditions only) field studies. Our study examines transects individually and autonomously with regional synoptic averages—at 400 km—to smooth out local diversity often due to anthropogenic impact and capture natural climate regional patterns with a substantial degree of common evolution due to common forcing^13^. This approach ensures minimization of the unavoidable uncertainty, that we acknowledge and thoroughly discussed in our paper (see Section “Limitations”) while focusing on a regional representation, a facet we have elaborated on in our supplementary material. As such, following the relatively coarse resolution of the global, publicly available driver datasets used here, our effort to comprehensively monitor coastlines through the unparalleled global vision of satellites introduces a novel and fresh perspective on waterline drivers and is rather a call for more detailed research to address the dominant components and their influence on the shoreline. We accompany our analysis with acknowledging the possible caveats which are inherent to the limitations of global datasets^14^.

The annual aggregates of waterline position also significantly reduce tide effects and several complex hydro-sedimentary short-term effects such as extremes, cumulative effects, and beach recovery^15^. As stated in our paper, and most of climate studies, our results should not be extrapolated to local scales; instead, they paint a comprehensive picture of the dominant drivers and the intricate connections between shoreline variability and climate modes at the global scale.

## Climate variability on waterline position: toward predictability

Contrary to Warrick et al.’ assertion, astronomical tides are independent from ENSO which is driven by internal Earth system dynamics, although some co-variability may generate overlap on long timescales^16^. However, our observational record of waterline position remains too limited to clearly attribute bi-decadal variability to this astronomical forcing. Our intention is not to capture the full evolution of the waterline position, but rather to show that the new complex version of ENSO used in our study^17^ (Warrick et al. refer to the canonical Niño3 4 index, an index that, while useful, is far too limited to capture the spatial diversity and temporal irregularity of the phenomenon), not only leaves discernible traces on the variability of the global waterline position at this specific interannual scale, but also allows to comprehend the chain of processes through which ENSO alternations through environmental conditions can lead to predictable coastal hazards in the Pacific basin (waterline is a proxy for both flooding and erosion^18^) and beyond. Warrick et al. argues that 25% of explained waterline variance explained by the climate ENSO model is weak when aggregated worldwide. We argue that this value is regionally larger around the Pacific and in the Intertropical band as we show statistically significant teleconnection beyond. Here, we examine the dominant drivers of the waterline, but also establish a direct link between the waterline and ENSO. This dispenses with the need to predict the drivers and allows a straightforward use of ENSO forecasts to inform waterline predictions^18^. While our paper clearly acknowledges that the influence of ENSO does not account for the majority of the signal (about 25% of the variance), the significant advantage here lies in its predictability. ENSO is a slow coupled mode of variability with significant predictability over long lead times, whereas SAM and NAO have a very short predictability potential due to their intrinsic atmospheric nature. This underscores the potential for meaningful predictive insights within the realm of ENSO’s impact on waterline variability, even if it does not encompass the entirety of the phenomenon.

## Relative influence of waves, sea level, and rivers on waterline position

The definition of wave-dominated (i.e., compared to only tidal dominance, following the wave-tide classification^19^) beaches deserves some perspective as, historically, sea level, waves and river effects on shoreline have not been analyzed together in the literature. The term wave-dominated implicitly, and in a process framework, refers to short storm scales rather than long interannual scales. Whereas wave predominance is generally the case for storm-dominated beaches, this is simply not the case for longer timescales where sea level and even rivers can drive the evolution. For instance, climate projections show that sea level^20^, and equally river flows^21^, are likely to experience substantial trends and changes in the long-term perspective, while waves show relatively weaker global trend^4^. At the interannual timescales considered in our paper, many studies show that regional sea level fluctuations play a dominant role on the coastal water level variability^22,23^ and therefore most likely on the waterline position.

Finally, we want to assure the authors of the Matters Arising that our global study is a first step forward, and we do not claim to do everything or be all-encompassing. The local and global communities with the triad—in-situ observation, modeling, and remote sensing—should work in synergy to construct a better knowledge of the evolution of our coastlines. This is the only way forward to address the important challenge we are all facing, towards a better holistic, interdisciplinary, and sustainability science.
